# Population Structure among *Mycobacterium tuberculosis* Isolates from Pulmonary Tuberculosis Patients in Colombia

**DOI:** 10.1371/journal.pone.0093848

**Published:** 2014-04-18

**Authors:** Teresa Realpe, Nidia Correa, Juan Carlos Rozo, Beatriz Elena Ferro, Verónica Gomez, Elsa Zapata, Wellman Ribon, Gloria Puerto, Claudia Castro, Luisa María Nieto, Maria Lilia Diaz, Oriana Rivera, David Couvin, Nalin Rastogi, Maria Patricia Arbelaez, Jaime Robledo

**Affiliations:** 1 Corporación para Investigaciones Biológicas, CIB, Medellín, Colombia; 2 Universidad Pontificia Bolivariana, Medellín, Colombia; 3 Centro Internacional de Entrenamiento e Investigaciones Médicas, CIDEIM, Cali, Colombia; 4 Instituto Nacional de Salud, Bogotá, Colombia; 5 Universidad del Cauca, Popayán, Colombia; 6 Universidad de Antioquia, Medellín, Colombia; 7 Centro Colombiano de Investigación en Tuberculosis, CCITB, Medellín, Colombia; 8 Universidad Industrial de Santander, Bucaramanga, Colombia; 9 WHO Supranational TB Reference Laboratory, TB & Mycobacteria Unit, Institut Pasteur de la Guadeloupe, Abymes Guadeloupe, France; Institut National de la Recherche Agronomique, France

## Abstract

**Background:**

Phylogeographic composition of *M. tuberculosis* populations reveals associations between lineages and human populations that might have implications for the development of strategies to control the disease. In Latin America, lineage 4 or the Euro-American, is predominant with considerable variations among and within countries. In Colombia, although few studies from specific localities have revealed differences in *M. tuberculosis* populations, there are still areas of the country where this information is lacking, as is a comparison of Colombian isolates with those from the rest of the world.

**Principal Findings:**

A total of 414 *M. tuberculosis* isolates from adult pulmonary tuberculosis cases from three Colombian states were studied. Isolates were genotyped using *IS6110*-restriction fragment length polymorphism (RFLP), spoligotyping, and 24-locus Mycobacterial interspersed repetitive units variable number tandem repeats (MIRU-VNTRs). SIT42 (LAM9) and SIT62 (H1) represented 53.3% of isolates, followed by 8.21% SIT50 (H3), 5.07% SIT53 (T1), and 3.14% SIT727 (H1). Composite spoligotyping and 24-locus MIRU- VNTR minimum spanning tree analysis suggest a recent expansion of SIT42 and SIT62 evolved originally from SIT53 (T1). The proportion of Haarlem sublineage (44.3%) was significantly higher than that in neighboring countries. Associations were found between *M. tuberculosis* MDR and SIT45 (H1), as well as HIV-positive serology with SIT727 (H1) and SIT53 (T1).

**Conclusions:**

This study showed the population structure of *M. tuberculosis* in several regions from Colombia with a dominance of the LAM and Haarlem sublineages, particularly in two major urban settings (Medellín and Cali). Dominant spoligotypes were LAM9 (SIT 42) and Haarlem (SIT62). The proportion of the Haarlem sublineage was higher in Colombia compared to that in neighboring countries, suggesting particular conditions of co-evolution with the corresponding human population that favor the success of this sublineage.

## Introduction

Tuberculosis (TB) continues to be a challenge to control. Although widespread and common efforts have had an impact in achieving declining numbers in global incidence for the first time in history, TB still causes 8.7 million new cases and 1.4 million deaths per year [1].

The worldwide population structure of *Mycobacterium tuberculosis* has been defined, linking specific lineages to human populations. Using comparative genomics and large sequence polymorphisms (LSPs), six phylogeographic lineages have been described and associated with human populations around the world [2]. For example, the East-Asian lineage is dominant in many countries of the Far East, while the Indo-Oceanic lineage occurs all around the Indian Ocean. The Euro-American lineage is clearly the most frequent lineage in Europe and the Americas. The relationships between these lineages and human populations are supported not only by studies with isolates from around the world, but also by the tendency of each lineage to cause the disease in populations in specific urban cosmopolitan settings [3]–[6].

Genotyping techniques based on repetitive elements such as restriction fragment length polymorphism (RFLP) using *IS6110* [7], spoligotyping of clustered repetitive interspersed short palindromic repeats (CRISPR) [8], and mycobacterial interspersed repetitive units variable number tandem repeats (MIRU-VNTRs) [9], have been used to support epidemiological studies as well as to define the population structure in *M. tuberculosis* [10]–[12]. Spoligotyping and MIRU-VNTR have replaced the standard *IS6110*-RFLP due to the ease of implementation and standardization, and the availability of international databases for making comparisons [10], [13], [14]. These latter techniques have demonstrated a concordance with the assignation of major lineages as defined by LSPs [15]–[17], despite being subject to convergent evolution [18].

Colombia is the third most populated country in Latin America. The country has nearly 47 million inhabitants and has changed from being mainly a rural population at the beginning of the 20^th^ century to a mostly urban population in the 21^th^ century. The increase in urban population together with crowding and poor living conditions in the outskirts of major cities maintain favorable conditions for TB transmission. Despite the country's efforts to control the disease, the estimated incidence is 34 per 100,000 individuals in the population, corresponding to approximately 16,000 new cases per year [19], which poses a major task for the public health control of the disease. The overall incidence of TB for Colombia hides the dissimilarities between regions, reflecting differences in control measures as well as differences in transmission dynamics. These situations in turn, should influence the relationship established between human and *M. tuberculosis* populations.

Several studies have demonstrated the distribution of *M. tuberculosis* lineages and sublineages in Latin American countries, confirming the overwhelming predominance of the Euro-American lineage but with considerable variation in the distribution of sublineages or clades between and within countries [20]–[25]. In Colombia, studies performed on few specific locations also show a predominance of Euro-American lineages with differences among localities [26], [27].

The aim of this study was to further assess the distribution of *M. tuberculosis* lineages and sublineages in Colombia and to gain a better understanding of the dynamics of the disease. *M. tuberculosis* isolates were obtained from patients with pulmonary tuberculosis from three different regions of Colombia. All isolates were genotyped by using *IS6110*-RFLP, spoligotyping, and 24-locus MIRU-VNTR. Then, associations between the main *M. tuberculosis* sublineages and the demographic and epidemiologic characteristics of patients were evaluated. The discriminatory power of the different genotyping methods was also calculated.

## Methods

### Ethics statement

All study procedures were approved by the Ethics Review Boards of the participating institutions who were in charge of recruiting the patients: Universidad de Antioquia, Centro Internacional de Entrenamiento e Investigaciones Médicas, CIDEIM, and Universidad del Cauca. All patients had a signed written consent previously approved by the ethics committee. When patients were less than 18 years old an informed written and signed consent was obtained with the additional approval and sign of one of the parents. All sign consents were kept in physical files locked under the custody of principal investigators to maintain the anonymity of patients. The study was also approved by regional and local health authorities in: Antioquia state and Medellin city, Valle del Cauca state and Cali city and Cauca state and Popayan city.

### Study population

*M. tuberculosis* isolates were obtained from index tuberculosis patients belonging to three cohorts followed in three different cities in Colombia (Medellín, Cali, and Popayan) from March 2005 to 2008. These patients were part of a previous study performed in the same cities, were we studied factors associated with TB transmission among household contacts of patients with pulmonary tuberculosis [28].

Index cases were included consecutively from urban populations in cohorts from Medellin and Cali, whereas the smallest cohort included cases from Popayan as well as from smaller towns in Cauca state. An index case was included if the patient was older than 15 years and had at least one household contact as described previously [28]. Index cases were initially diagnosed based on clinical symptoms, signs, and chest-X rays, and confirmed by acid-fast bacilli (AFB) sputum examination using the Zielh-Nelsen stain, at the local health facility. A second sputum specimen was processed and cultured at the research laboratory designated in each city. Sputum samples were decontaminated with NaOH and N-acetyl-L-cysteine [29], cultured on an MGIT system (MGIT 960®) and/or solid Lowenstein-Jensen (LJ) culture media. Identification of AFB-positive cultures was performed by phenotypic methods such as niacin, nitrate and 68°C catalase tests [29]. Drug susceptibility testing for first line anti-TB drugs was performed using the proportion method in LJ [29]. *M. tuberculosis* isolates were frozen in 50% glycerol at −70°C until use. One isolate obtained from one AFB-positive smear sputum per patient was used for genotyping.

### *M. tuberculosis* genotyping

Isolates were genotyped using spoligotyping, *IS6110*-RFLP, and 24-locus MIRU-VNTRs. For *IS6110*-RFLP genotyping, a standard protocol was used following international recommendations, which included a DNA extraction protocol [7], [30]. Spoligotyping was performed following standard procedures [8], using a commercial source for membranes and reagents (Isogen Life Science, De Meern, the Netherlands).

MIRU-VNTR genotyping was performed using polymerase chain reaction (PCR) amplification of a standard set of 24 MIRU-VNTR loci with primers specific for the flanking regions of each VNTR region, and the detection of amplified PCR products was carried out by electrophoresis. From the gel images, the corresponding MIRU-VNTR bands were interpreted as copy numbers based on a reference table [9]. Two of the participating laboratories took part of the first and second multicenter proficiency studies of the Global Network for the Molecular Surveillance of Tuberculosis using MIRU-VNTR genotyping [31].

The role of participating laboratories was as follows: Mycobacteriology laboratory at University of Cauca in charge of culturing and identifying *M. tuberculosis* from patients in Popayan and surrounding towns (Cauca state). Mycobacteriology laboratory at Cideim was in charge of culturing and identifying *M. tuberculosis* from patients in Cali (Valle state) and performed 24-locus MIRU-VNTR in those isolates. Mycobacteriology laboratory at National Institute of Health in Bogota was in charge of performing drug susceptibility tests and genotyping by IS6110-RFLP, spoligotyping and 24-locus MIRU-VNTR to isolates from Cauca and Valle states. Mycobacteriology laboratory at CIB performed culture and identification of isolates from patients from Medellin (Antioquia state) as well as genotyping using IS6110-RFLP, spoligotyping and 24-locus MIRU-VNTR.

### Clustering analysis, allelic diversity, and discriminatory power

*IS6110*-RFLP, spoligotyping, and 24-locus MIRU-VNTRs results were analyzed using the BioNumerics software version 6.6 (Applied Maths. Sint-Martens-Latem, Belgium) to establish the relationships between different isolates of *M. tuberculosis*. Patterns of *IS6110*-RFLP were digitized and similarities were calculated using the Dice coefficient. MIRU-VNTR and spoligotyping data were entered as character type and analyzed using the categorical coefficient. Similarity trees and dendrograms were calculated using the unweighted pair group method with arithmetic averages (UPGMA). A cluster was defined as two or more *M. tuberculosis* isolates with identical patterns. The MIRU-VNTR allelic diversity (*h*) at a given locus was calculated as *h* = 1−∑ *xi*2 [(*n*/*n*−1)], where *xi* is the frequency of the *i*th allele at the locus and *n* is the number of isolates [9], [32]. To determine the discriminatory power (DP) of each genotyping method or a combination thereof, the Hunter-Gaston Discriminatory Index (HGDI) was calculated [33].

Minimum spanning trees (MST) were created using the Bionumerics software (Version 6.60) to explore the evolutionary relationship among spoligotyping and 24-locus MIRU-VNTR isolates. Spoligoforest trees were drawn to determine the parent-to-descendant spoligotypes in the group of isolates studied using the Fruchterman-Reingold algorithm and a hierarchical layout using the SpolTools software [http://www.emi.unsw.edu.au/spolTools, [34]], and reshaped and colored using the GraphViz software [http://www.graphviz.org].

### Lineage assignation and comparison with an international database

Spoligotypes in binary format were converted to an octal code for comparison with the SITVIT2 proprietary database of Institut Pasteur de la Guadeloupe, which is an updated version of the previously released SpolDB4 and SITVITWEB databases [10], [13]. At the time of the analysis, SITVIT2 contained genotyping information on about 110.000 *M. tuberculosis* clinical isolates from 160 countries of origin. In this database, a Spoligotype International Type (SIT) represents a spoligotyping pattern shared by 2 or more patient isolates, as opposed to “orphan,” which does not match with another pattern in the SITVIT2 database. Major phylogenetic clades were assigned according to spoligotype signatures and using revised SpolDB4/SITVITWEB rules [10], [13]. The sublineage distribution in cities from this study was also compared with those from two other cities of Colombia (Buenaventura and Bogotá), for which data were available in the SITVIT2 database. We also compared the distribution of the predominant SITs in the present study with the available data for 3 neighboring countries (Venezuela, Brazil, and Peru) in the SITVIT2 database.

Descriptive statistics were used to show the distribution of lineages and SITs per cohort of patients. STATA version 12 (STATA Corp. USA) was used for statistical analysis. Association of clades and SITs with demographic and epidemiological characteristics (human immunodeficiency virus [HIV] serology, sex), susceptibility to first-line drugs, and number of *IS6110*-RFLP copies, as well as differences in distribution according to cohorts, were calculated using Pearson's Chi-square test when more than 80% of the data had values greater than 5 and Fisher's Exact Test for the remaining data with smaller values (where at least 20% of data had values less than 5).

All study procedures and written consent forms were approved by the Ethics Review Boards of the participating institutions.

## Results

Four hundred and fourteen *M. tuberculosis* isolates were studied from index cases included in three cohorts followed in three different Colombian cities for a period of three years (2005 to 2008). The median age of the patients was 39.1 years (range 15 to 96 years), and 42.8% of them were female. Bacillus Calmette-Guerin (BCG) vaccination was confirmed in 75.6% of patients and 1.8% were sero-positive for HIV. Most of the isolates (75.1%) were pan-susceptible to anti-TB drugs, 12.1% exhibited some drug resistance, and 4.6% were resistant to both isoniazid and rifampin (multi-drug resistant, MDR).

A total of 84 spoligotypes were identified; these included 20 orphan patterns that have not yet been reported to the SITVIT2 database (Table 1). The other 64 patterns matched a preexisting shared type in the database (50/64 SITs containing 374 isolates) or created a new shared type (14/64 SITs containing 20 isolates) within this study or after a match with a previously reported orphan in the SITVIT2 database (Table 2). Furthermore, 25 out of 64 pre-existing SITs containing 355 isolates were clustered (2 to 124 isolates per cluster), corresponding to 85.75% of all isolates. The number of unclustered isolates was 59 (39 isolates with unique SITs plus 20 orphan isolates) out of 414, or 14.25%.

**Table 1:** Orphan strains (n = 20) and corresponding spoligotyping defined lineages/sublineages recorded among a total of 414 *M. tuberculosis* strains from Colombian patients.

IsoNumber	Year	Strain	Spoligotype Description	Octal code	Lineage*	Label	MIRU24	24-MIT	Drug R**	Isolation city	Sex/Age	IS6110 RFLP	HIV
COL012005100005	2005	00005	▪▪▪▪▪▪▪▪□□▪▪▪▪□▪▪▪▪▪▪▪▪▪□□□□□□□□□□□□▪▪▪□▪▪▪	776367770000731	Unknown	o01	222325143322424352314233	o	1	Medellin	M/24	8	(-)
COL012005100028	2005	00028	▪▪□▪▪▪▪▪▪□▪▪▪▪□▪▪▪▪▪□□□□▪▪▪▪▪▪▪▪□□□□▪▪□▪▪▪▪	677367607760671	LAM1	o02	223226193321224262434132	o	1	Medellin	F/40	9	(-)
COL012005300032	2005	00032	▪▪▪▪▪▪▪□▪□▪▪▪□▪▪▪▪▪▪▪▪▪▪▪▪▪▪▪▪▪▪□□□□▪▪▪▪▪▪▪	775357777760771	T1	o03	222325153325124522224233	191	3 (INH, SM)	Medellin	F/83	7	(-)
COL012005100066	2005	00066	▪▪▪▪▪▪▪▪▪▪▪▪▪□▪▪▪▪▪▪▪▪▪▪▪▪▪□□□□▪□□□□▪▪▪▪▪▪▪	777757777020771	H3	o04	225325153323323563224433	o	1	Medellin	F/26	8	(-)
COL012005100068	2005	00068	▪▪▪▪▪□▪▪▪▪▪▪▪▪▪▪▪▪▪▪□□□□▪□▪▪▪▪▪▪□□□□▪▪▪▪▪▪▪	767777605760771	LAM9	o05	124326153220224252334132	o	1	Medellin	F/17	10	(-)
COL012005100071	2005	00071	▪□▪▪□▪▪▪▪□▪▪▪□□▪▪▪▪▪▪▪▪▪▪▪▪▪▪▪▪▪□□□□▪▪□▪▪□▪	557347777760661	T1	o06	222325153325124522224233	191	1	Medellin	M/41	6	(-)
COL012005100081	2005	00081	▪▪▪▪▪▪▪▪▪□▪▪▪□▪▪▪▪▪▪▪▪▪▪▪▪▪□□□□▪□□□□▪▪▪▪▪▪▪	777357777020771	H3	o07	225325153323323373124433	o	1	Medellin	M/35	8	(-)
COL012005000161	2005	00161	▪▪▪▪▪▪▪▪▪□▪▪▪□▪▪▪▪▪▪□□□□▪▪▪▪▪▪▪▪□□□□▪▪□▪▪▪▪	777357607760671	LAM9	o08	123326153216224274434132	o	0	Medellin	M/34	10	(-)
COL012005100178	2005	00178	▪▪▪▪▪▪▪▪▪□▪▪▪▪□□□▪□▪▪▪▪▪▪▪▪▪▪▪▪▪□□□□▪▪□▪▪▪▪	777361377760671	T4	o09	223225153324324453324233	o	1	Medellin	M/46	9	(-)
COL012006100203a	2006	00203a	▪▪▪□□□□□□□□□□▪▪▪▪□▪▪▪▪▪▪▪□▪▪▪▪▪▪□□□□▪▪▪▪▪▪▪	700036775760771	X3	o10	224325153322323660434433	o	1	Cali	F/23	2	(-)
COL012005000246b	2005	00246b	▪▪▪▪▪▪□□□▪▪▪▪▪▪▪▪▪▪▪▪▪□▪▪▪▪▪▪▪▪▪▪▪▪▪▪□□▪▪▪▪	770777757777471	AFRI	o11	226224243521445413243434	o	0	Medellin	M/60	6	(-)
COL012006100302	2006	00302	▪▪▪▪▪▪▪□□▪▪▪▪▪▪▪▪▪▪▪□□□□▪▪▪▪▪▪▪▪□□□□▪▪▪▪▪▪▪	774777607760771	LAM9	o12	124326153220224162234132	o	1	Cali	M/42	11	(-)
COL012006100313	2006	00313	▪▪▪▪▪□▪▪▪▪▪▪▪▪▪▪▪▪▪▪▪▪▪□▪▪▪▪▪▪□▪□□□□▪▪▪▪▪▪▪	767777767720771	H3	o13	125325153323333563234434	o	1	Medellin	F/22	8	(-)
COL012006100340	2006	00340	▪▪▪▪▪▪▪▪▪▪▪▪▪▪▪▪▪▪□▪▪▪▪▪▪□□□□□□▪□□□□▪▪▪□▪▪▪	777777374020731	H1	o14	225325153323233474234424	197	1	Medellin	M/27	8	(-)
COL012006000348	2006	00348	▪▪▪▪▪▪▪▪▪▪▪▪▪□□□□▪□□▪▪▪▪▪▪▪▪▪▪□▪□□□□▪▪▪▪▪▪▪	777741177720771	H3	o15	225313153323323573222433	209	0	Medellin	M/41	11	(-)
COL012006100408	2006	00408	▪▪▪□□▪▪▪▪▪▪▪□▪▪▪▪▪▪▪▪▪▪▪▪□□□□□□▪□□□□▪▪▪□▪▪▪	717737774020731	H1	o16	225325153324333473334423	204	1	Medellin	M/26	12	(-)
COL012006100422	2006	00422	▪□▪▪▪▪▪▪□□▪▪▪▪▪▪▪▪▪▪▪□□□□▪▪▪▪▪▪□□□□□▪▪▪□▪▪▪	576377703740731	S	o17	252325153324424352214234	o	1	Medellin	F/45	13	(-)
COL012006000473b	2006	00473b	▪▪▪▪▪▪▪▪□□▪▪▪▪▪▪▪▪▪▪▪□□□□□▪▪▪▪▪□□□□□▪▪▪□▪▪▪	776377701740731	S	o18	222325163324324522224233	226	0	Popayan	M/60	9	(-)
COL012006100486b	2006	00486b	▪▪□▪▪▪▪▪▪▪▪▪□▪▪▪▪▪▪▪□□□□□▪▪▪▪▪▪▪□□□□▪▪▪▪▪▪▪	677737603760771	T3	o19	222226153221224482334233	o	1	Popayan	M/91	8	(-)
COL012007100524	2007	00524	□□□□□□□□□□□□□□□□▪▪▪▪▪▪▪▪□□□□□□□□□□□□▪▪▪□▪▪▪	000003770000731	Unknown	o20	232325253324424352314234	o	1	El tambo	F/36	10	(-)

* Lineage designations for orphan patterns were done manually as Expert-based interpretations using revised SpolDB4 rules.

** Drug resistance (Drug R) information is shown as: 0, unknown; 1, pansusceptible; 2, MDRTB, i.e., combined resistance to INH-RIF (with or without resistance to other drugs); 3, any other resistance(s); 4, proven XDRTB.

**Table 2:** Description of 64 shared-types (SITs; n = 394 isolates) and corresponding spoligotyping defined lineages/sublineages starting from a total of 414 *M. tuberculosis* strains isolated from Colombian patients.

SIT*	Spoligotype Description	Octal Code	Nb in study	% in this study	% in study vs. Database	Lineage**	Clustered vs. Unique patterns***
2	□□□□□□□□□□□□□□□□□□□□□□□□▪□□□□□□▪□□□□▪▪▪▪▪▪▪	000000004020771	2	0.48	0.51	H2	Clustered
3	□□□□□□□□□□□□□□□□□□□□□□□□▪▪▪▪▪▪□▪□□□□▪▪▪▪▪▪▪	000000007720771	1	0.24	0.95	H3	Unique
4	□□□□□□□□□□□□□□□□□□□□□□□□▪▪▪▪▪▪▪▪□□□□▪▪▪▪▪▪▪	000000007760771	1	0.24	0.28	Unknown	Unique
8	▪□□□□□□□□□□□□▪▪▪▪▪▪▪▪▪▪▪▪▪▪▪□□□□▪□▪▪▪▪▪▪▪▪▪	400037777413771	1	0.24	0.55	EAI5	Unique
17	▪▪□▪▪▪▪▪▪▪▪▪□▪▪▪▪▪▪▪□□□□▪▪▪▪▪▪▪▪□□□□▪▪▪▪▪▪▪	677737607760771	2	0.48	0.3	LAM2	Clustered
20	▪▪□▪▪▪▪▪▪▪▪▪▪▪▪▪▪▪▪▪□□□□▪▪▪▪▪▪▪▪□□□□▪▪▪▪▪▪▪	677777607760771	7	1.69	0.86	LAM1	Clustered
33	▪▪▪▪▪▪▪▪□□□▪▪▪▪▪▪▪▪▪□□□□▪▪▪▪▪▪▪▪□□□□▪▪▪▪▪▪▪	776177607760771	3	0.72	0.27	LAM3	Clustered
37	▪▪▪▪▪▪▪▪▪▪▪▪□▪▪▪▪▪▪▪▪▪▪▪▪▪▪▪▪▪▪▪□□□□▪▪▪▪▪▪▪	777737777760771	2	0.48	0.43	T3	Clustered
42	▪▪▪▪▪▪▪▪▪▪▪▪▪▪▪▪▪▪▪▪□□□□▪▪▪▪▪▪▪▪□□□□▪▪▪▪▪▪▪	777777607760771	124	29.95	3.78	LAM9	Clustered
45	▪▪▪▪▪▪▪▪▪▪▪▪▪▪▪▪▪▪▪▪▪▪▪□▪□□□□□□▪□□□□▪▪▪▪▪▪▪	777777764020771	6	1.45	5.66	H1	Clustered
47	▪▪▪▪▪▪▪▪▪▪▪▪▪▪▪▪▪▪▪▪▪▪▪▪▪□□□□□□▪□□□□▪▪▪▪▪▪▪	777777774020771	2	0.48	0.13	H1	Clustered
50	▪▪▪▪▪▪▪▪▪▪▪▪▪▪▪▪▪▪▪▪▪▪▪▪▪▪▪▪▪▪□▪□□□□▪▪▪▪▪▪▪	777777777720771	34	8.21	1.02	H3	Clustered
53	▪▪▪▪▪▪▪▪▪▪▪▪▪▪▪▪▪▪▪▪▪▪▪▪▪▪▪▪▪▪▪▪□□□□▪▪▪▪▪▪▪	777777777760771	21	5.07	0.34	T1	Clustered
62	▪▪▪▪▪▪▪▪▪▪▪▪▪▪▪▪▪▪▪▪▪▪▪▪▪□□□□□□▪□□□□▪▪▪□▪▪▪	777777774020731	97	23.43	17.7	H1	Clustered
64	▪▪▪▪▪▪▪▪▪▪▪▪▪▪▪▪▪▪▪▪□□□□▪▪▪▪□▪▪▪□□□□▪▪▪▪▪▪▪	777777607560771	1	0.24	0.27	LAM6	Unique
86	▪▪▪▪▪▪▪▪▪▪▪▪▪▪▪▪▪▪▪▪▪□▪▪▪▪▪▪▪▪▪▪□□□□▪▪▪▪▪▪▪	777777737760771	1	0.24	1.09	T1	Unique
91	▪▪▪□□□□□□□□□□▪▪▪▪□▪▪▪▪▪▪▪▪▪▪▪▪▪▪□□□□▪▪▪▪▪▪▪	700036777760771	5	1.21	1.98	X3	Clustered
93	▪▪▪▪▪▪▪▪▪▪▪▪□▪▪▪▪▪▪▪□□□□▪▪▪▪▪▪▪▪□□□□▪▪▪▪▪▪▪	777737607760771	1	0.24	0.28	LAM5	Unique
102	▪▪▪▪▪▪▪▪▪▪▪▪□□□□▪▪▪▪▪▪▪▪▪▪▪▪▪▪▪▪□□□□▪▪▪▪▪▪▪	777703777760771	1	0.24	1.37	T	Unique
106	▪▪▪▪▪▪▪▪□□□▪▪▪▪▪▪▪▪□□□□□□□□□□□□□□□□□□□▪▪▪▪▪	776177400000171	1	0.24	0.76	Unknown	Unique
124	▪▪▪▪▪▪▪▪▪▪▪▪▪▪▪▪▪▪▪▪▪▪▪▪▪▪▪▪▪▪□□□□□□▪▪▪▪▪▪▪	777777777700771	2	0.48	3.03	Unknown	Clustered
130	▪▪▪▪▪▪▪▪□□□▪▪▪▪▪▪▪▪▪□□□□▪▪▪▪▪▪▪▪□□□□▪▪▪□▪▪▪	776177607760731	1	0.24	0.93	LAM3	Unique
162	▪▪▪▪▪▪▪▪▪▪▪▪▪▪▪▪▪▪▪▪□□□□▪▪▪▪▪▪▪▪□□□□▪▪□▪▪▪▪	777777607760671	1	0.24	3.23	LAM9	Unique
167	▪▪▪▪▪▪▪▪▪▪▪▪▪▪▪▪▪▪▪▪▪▪▪▪▪▪▪▪▪□▪▪□□□□▪▪▪▪▪▪▪	777777777660771	1	0.24	1.37	T1	Unique
207	▪▪▪▪▪□▪▪▪▪▪▪▪▪▪▪▪▪▪▪▪▪▪▪▪▪▪▪▪▪□▪□□□□▪▪▪▪▪▪▪	767777777720771	8	1.93	25.81	H3	Clustered
230	▪▪▪▪▪▪▪▪▪▪▪▪▪▪▪▪▪▪▪▪□□□□□▪▪▪▪▪▪▪□□□□▪▪▪▪▪▪▪	777777603760771	1	0.24	5.56	LAM9	Unique
237	▪▪▪▪▪▪▪▪▪▪▪▪▪▪▪▪▪▪▪▪▪▪▪▪▪▪▪▪▪▪□□□□□□□□□□□□□	777777777700000	1	0.24	0.93	Unknown	Unique
254	▪▪▪▪▪▪▪▪▪▪▪▪▪▪□□□□□□□□□□▪▪▪▪▪▪▪▪□□□□▪▪▪▪▪▪▪	777760007760771	1	0.24	0.57	T5-RUS1	Unique
316	▪▪▪▪▪▪▪▪▪▪▪▪▪▪▪▪▪▪▪▪▪▪▪▪□□□□□□□▪□□□□▪▪▪□▪▪▪	777777770020731	1	0.24	2.13	H3	Unique
379	▪▪▪▪▪▪▪▪▪▪▪▪▪□□□□▪□□□□□▪▪▪▪▪▪▪▪▪□□□□▪▪▪▪▪▪▪	777741017760771	2	0.48	9.52	Unknown	Clustered
384	▪▪▪▪▪▪▪▪▪▪▪▪▪▪▪▪▪▪▪▪□▪▪▪▪□□□□□□▪□□□□▪▪▪▪▪▪▪	777777674020771	1	0.24	11.11	H1	Unique
447	▪▪▪▪▪▪▪▪▪▪▪▪▪▪□□□□□▪▪▪▪▪▪▪▪▪▪▪▪▪□□□□▪▪▪▪▪▪▪	777760377760771	1	0.24	12.5	T1	Unique
727	▪▪▪▪▪▪▪▪▪▪▪▪□▪▪▪▪▪▪▪▪▪▪▪▪□□□□□□▪□□□□▪▪▪□▪▪▪	777737774020731	13	3.14	34.21	H1	Clustered
753	▪□□▪▪▪▪▪▪▪▪▪▪▪▪▪▪▪▪▪□□□□▪▪▪▪▪▪▪▪□□□□▪▪▪▪▪▪▪	477777607760771	1	0.24	5	LAM1	Unique
881	▪▪▪▪▪▪▪▪□□▪▪▪▪▪▪▪▪▪▪▪▪▪▪□□□□□□□□□□□□▪▪▪□▪▪▪	776377770000731	6	1.45	24	Unknown	Clustered
1243	▪▪▪▪▪▪▪▪▪□▪▪▪▪▪▪▪▪▪▪▪▪▪▪▪▪▪▪▪▪□▪□□□□▪▪▪▪▪▪▪	777377777720771	1	0.24	6.25	H3	Unique
1277	▪▪▪▪▪▪▪▪▪▪▪▪▪▪▪▪▪▪□▪□□□□▪▪▪▪▪▪▪▪□□□□▪▪▪▪▪▪▪	777777207760771	1	0.24	3.85	LAM9	Unique
1283	▪▪▪▪▪▪▪▪▪▪▪▪▪▪▪▪▪▪▪▪▪▪▪▪▪▪▪□□□□▪□□□□▪▪▪▪▪▪▪	777777777020771	1	0.24	11.11	H3	Unique
1486	□▪▪□□□□□□□□□□▪▪▪▪□▪▪▪▪▪▪▪▪▪▪▪▪▪▪□□□□▪▪▪▪▪▪▪	300036777760771	1	0.24	33.33	X3	Unique
1533	▪▪▪▪▪▪▪▪▪▪▪▪▪▪□□▪▪▪▪▪▪▪▪▪▪▪▪▪▪□▪□□□□▪▪▪▪▪▪▪	777763777720771	2	0.48	28.57	H3	Clustered
1536	▪▪▪▪▪▪▪▪□□▪▪▪▪▪▪▪▪▪▪□□□□▪▪▪▪▪▪▪▪□□□□▪▪▪▪▪▪▪	776377607760771	1	0.24	4.76	LAM9	Unique
1561	▪▪▪▪▪▪▪▪▪▪▪▪▪▪▪▪▪▪▪▪▪▪▪▪▪□□□□□□▪□□□□□▪▪▪▪▪▪	777777774020371	1	0.24	20	H1	Unique
1804	▪▪▪▪▪▪▪▪▪▪▪▪▪▪▪▪▪▪▪□▪▪▪▪▪▪▪▪▪▪□▪□□□□▪▪▪▪▪▪▪	777777577720771	2	0.48	28.57	H3	Clustered
1832	▪▪▪▪▪▪▪▪▪▪▪▪▪▪▪□▪▪▪▪□□□□▪▪▪▪▪▪▪▪□□□□▪▪▪▪▪▪▪	777773607760771	1	0.24	5	LAM9	Unique
2025	▪▪▪▪▪▪▪▪▪▪▪▪▪▪▪▪▪▪▪▪▪▪▪▪▪▪▪▪▪▪□□□□□□□▪▪▪▪▪▪	777777777700371	1	0.24	9.09	T	Unique
2114	▪▪▪▪▪▪▪▪▪▪▪▪▪▪□□□▪▪▪▪▪▪▪▪▪▪▪▪▪▪▪□□□□▪▪▪▪▪▪▪	777761777760771	1	0.24	14.29	T1	Unique
2497	▪▪□▪▪▪▪▪▪▪▪□▪▪▪▪▪▪▪▪□□□□▪▪▪▪▪▪▪▪□□□□▪▪▪▪▪▪▪	677677607760771	1	0.24	16.67	LAM1	Unique
2865	▪▪▪▪▪▪▪▪▪▪▪▪▪▪▪▪▪▪▪▪□□□□▪▪▪▪□□▪▪□□□□▪▪▪▪▪▪▪	777777607460771	1	0.24	25	LAM9	Unique
2940	▪▪▪▪▪▪▪▪▪▪▪▪▪▪▪▪▪▪▪▪□□□□▪▪▪▪▪▪▪▪□□□□▪□□▪▪▪▪	777777607760471	2	0.48	28.57	LAM9	Clustered
3010	▪▪▪▪▪▪▪▪□□▪▪▪▪▪▪▪▪▪▪▪▪▪▪▪▪▪▪▪▪▪□□□□□▪▪▪□▪▪▪	776377777740731	3	0.72	33.33	S	Clustered
3018*	▪▪▪▪▪▪▪□□□□□□□□▪▪□▪▪▪▪▪▪▪▪▪▪▪▪▪▪□□□□▪▪▪▪▪▪▪	774006777760771	1	0.24	50	X1	Unique
3019*	▪□▪▪▪▪▪▪▪▪▪▪□▪▪▪▪▪▪▪□□□□▪▪▪▪▪▪▪▪□□□□▪▪▪▪▪▪▪	577737607760771	1	0.24	25	LAM5	Unique
3020*	▪▪▪▪▪▪▪▪▪▪▪▪▪▪▪▪▪▪▪▪□□□□▪▪▪□▪▪▪▪□□□□▪▪▪▪▪▪▪	777777607360771	1	0.24	25	LAM9	Unique
3022*	▪▪▪□□□□□□□□□▪▪▪▪▪▪▪▪▪▪▪▪▪□□□□□□▪□□□□▪▪▪□▪▪▪	700077774020731	3	0.72	100	H1	Clustered
3023*	▪▪▪□□▪▪▪□□□▪▪▪▪▪▪▪▪▪□□□□▪▪▪▪▪▪▪▪□□□□▪▪▪▪▪▪▪	716177607760771	1	0.24	50	LAM3	Unique
3039*	▪▪▪▪▪▪▪▪▪▪▪▪▪▪□□□▪□▪▪▪▪▪▪▪▪▪▪▪▪▪□□□□▪▪▪▪▪▪▪	777761377760771	3	0.72	75	T4	Clustered
3040*	▪▪▪▪▪▪▪▪▪□▪▪▪▪□▪▪▪▪▪□□□□▪▪▪▪▪▪▪▪□□□□▪▪□▪▪▪▪	777367607760671	1	0.24	50	LAM9	Unique
3041*	▪▪□▪▪▪▪▪▪▪▪▪▪▪▪▪▪▪▪▪□□□□▪▪▪▪▪▪▪▪□□□□▪▪▪□□□□	677777607760700	2	0.48	66.67	LAM	Clustered
3042*	▪▪▪▪▪▪▪▪▪▪▪▪▪▪▪▪▪▪▪▪□▪▪▪▪□□□□□□▪□□□□▪▪▪□▪▪▪	777777674020731	1	0.24	50	H1	Unique
3043*	▪▪▪▪□□□▪▪▪▪▪▪▪▪▪▪▪▪▪▪▪▪▪▪□□□□□□▪□□□□▪▪▪▪▪▪▪	743777774020771	1	0.24	50	H1	Unique
3044*	▪▪▪▪▪▪▪▪▪▪▪▪▪▪▪▪▪▪▪▪□□□□▪▪▪▪▪▪□□□□□□□□□▪▪▪▪	777777607700071	2	0.48	100	Unknown	Clustered
3045*	▪▪□▪▪▪▪▪▪▪▪▪□▪▪▪▪▪▪▪□□□□□□□□□□□□□□□□□□□□□□□	677737600000000	1	0.24	50	Unknown	Unique
3311*	□□□□□□▪▪□□□▪▪▪▪▪▪▪▪▪□□□□▪▪▪▪▪▪▪▪□□□□▪▪▪▪▪▪▪	006177607760771	1	0.24	33.33	LAM3	Unique
3462*	▪▪▪▪▪▪▪▪▪▪▪▪▪□□□□□□▪▪▪▪▪▪▪▪▪▪▪▪▪□□□□▪▪▪▪▪▪▪	777740377760771	1	0.24	50	T1	Unique

* newly created SITs due to 2 or more strains belonging to an identical new pattern within this study or after a match with an orphan in the database; SIT designations followed by number of strains: 3018* this study n = 1, USA n = 1; 3019* this study n = 1, BRA n = 2, SEN n = 1; 3020* this study n = 1, FXX n = 1, GNB n = 2; 3022* this study n = 3; 3023* this study n = 1, CUB n = 1; 3039* this study n = 3, COL n = 1; 3040* this study n = 1, ESP n = 1; 3041* this study n = 2, BRA n = 1; 3042* this study n = 1, VEN n = 1; 3043* this study n = 1, DEU n = 1; 3044* this study n = 2; 3045* this study n = 1, VEN n = 1; 3311* this study n = 1,COL n = 2; 3462* this study n = 1, FXX n = 1;

** Lineage designations according to SITVIT2 using revised SpolDB4 rules; “Unknown” designates patterns with signatures that do not belong to any of the major lineages described in the database.

*** Clustered strains correspond to a similar spoligotype pattern shared by 2 or more strains “within this study”; as opposed to unique strains harboring a spoligotype pattern that does not match with another strain from this study. Unique strains matching a preexisting pattern in the SITVIT2 database are classified as SITs, whereas in case of no match, they are designated as “orphan” (see Table 1).

SIT 42 (LAM9) with 124 isolates and SIT 62 (H1) with 97 isolates represented 29.9% and 23.4% of the total isolates, respectively (Table 2). These two SITs accounted for 3.78% and 17.7% of the isolates when compared with the total number of isolates in the SITVIT2 database, and together represented more than 10% of the isolates in South America, North America, and Southern Europe. In contrast, SIT207 (H3) with 8 isolates and SIT727 (H1) with 13 isolates represented 25.8% and 34.2% of the isolates in the SITVIT2 database; these SITs have been reported mostly in South America and North America (Table 3, see also table S3 for comparison of sublineages distribution with neighboring countries).

**Table 3:** Description of clusters containing 5 or more isolates in this study, and their worldwide distribution in the SITVIT2 database.

SIT (Lineage) Octal Number Spoligotype Description	Octal Number	Number (%) in study	% in study vs. Database	Distribution in Regions with ≥3% of a given SIT *	Distribution in countries with ≥3% of a given SIT **
20 (LAM1) 677777607760771 ▪▪□▪▪▪▪▪▪▪▪▪▪▪▪▪▪▪▪▪□□□□▪▪▪▪▪▪▪▪□□□□▪▪▪▪▪▪▪	677777607760771	7 (1.69)	0.86	AMER-S 28.48, AMER-N 19.61, EURO-W 11.34, AFRI-S 10.97, EURO-S 9.74, CARI 5.3, AFRI-E 4.32	USA 19.61, BRA 18.13, NAM 7.65, PRT 6.04, FXX 6.04, VEN 5.18, ZAF 3.33, HTI 3.21, ESP 3.08
42 (LAM9) 777777607760771 ▪▪▪▪▪▪▪▪▪▪▪▪▪▪▪▪▪▪▪▪□□□□▪▪▪▪▪▪▪▪□□□□▪▪▪▪▪▪▪	777777607760771	124 (29.95)	3.78	AMER-S 29.77, AMER-N 12.1, EURO-S 11.55, EURO-W 9.66, AFRI-N 8.74, EURO-N 3.81, AMER-C 3.63, AFRI-E 3.63, AFRI-S 3.23	BRA 12.22, USA 12.1, COL 7.77, MAR 7.19, ITA 6.67, FXX 5.15, ESP 3.41, VEN 3.38, ZAF 3.23
45 (H1) 777777764020771 ▪▪▪▪▪▪▪▪▪▪▪▪▪▪▪▪▪▪▪▪▪▪▪□▪□□□□□□▪□□□□▪▪▪▪▪▪▪	777777764020771	6 (1.45)	5.66	EURO-W 24.53, CARI 24.53, AMER-S 16.04, EURO-S 11.32, AMER-N 9.43, EURO-N 5.66	AUT 16.98, MTQ 13.21, USA 9.43, ITA 8.49, BRA 8.49, GLP 7.55, COL 7.55, DEU 3.77
50 (H3) 777777777720771 ▪▪▪▪▪▪▪▪▪▪▪▪▪▪▪▪▪▪▪▪▪▪▪▪▪▪▪▪▪▪□▪□□□□▪▪▪▪▪▪▪	777777777720771	34 (8.21)	1.02	AMER-S 18.02, EURO-W 17.87, AMER-N 17.87, EURO-S 11.74, EURO-E 5.62, EURO-N 4.35, AFRI-N 4.32, AFRI-S 4.11, CARI 3.54, ASIA-W 3.27	USA 17.84, BRA 7.18, FXX 7.0, AUT 6.19, ITA 5.53, ESP 5.53, PER 4.81, ZAF 4.11, CZE 3.72
53 (T1) 777777777760771 ▪▪▪▪▪▪▪▪▪▪▪▪▪▪▪▪▪▪▪▪▪▪▪▪▪▪▪▪▪▪▪▪□□□□▪▪▪▪▪▪▪	777777777760771	21 (5.07)	0.34	EURO-W 16.1, AMER-N 13.88, AMER-S 12.39, EURO-S 9.68, ASIA-W 7.52, EURO-N 5.49, AFRI-S 5.11, AFRI-E 4.64, ASIA-E 4.36, AFRI-N 3.62, EURO-E 3.36, AMER-C 3.33	USA 13.58, FXX 8.11, ITA 5.49, BRA 5.28, ZAF 5.0, TUR 3.57, AUT 3.52, CHN 3.18
62 (H1) 777777774020731 ▪▪▪▪▪▪▪▪▪▪▪▪▪▪▪▪▪▪▪▪▪▪▪▪▪□□□□□□▪□□□□▪▪▪□▪▪▪	777777774020731	97 (23.43)	17.7	AMER-S 36.5, EURO-W 16.06, EURO-S 12.41, AMER-N 12.41, AFRI-E 3.65, AFRI-W 3.29	COL 34.12, USA 12.41, FXX 12.04, ITA 7.48, ESP 3.83, GMB 3.1
91 (X3) 700036777760771 ▪▪▪□□□□□□□□□□▪▪▪▪□▪▪▪▪▪▪▪▪▪▪▪▪▪▪□□□□▪▪▪▪▪▪▪	700036777760771	5 (1.21)	1.98	AMER-N 49.8, AMER-S 22.13, CARI 13.83, EURO-S 6.32, EURO-N 4.35	USA 46.64, HTI 11.46, PER 9.88, ESP 6.32, COL 4.74, GUF 3.95, CAN 3.16
207 (H3) 767777777720771 ▪▪▪▪▪□▪▪▪▪▪▪▪▪▪▪▪▪▪▪▪▪▪▪▪▪▪▪▪▪□▪□□□□▪▪▪▪▪▪▪	767777777720771	8 (1.93)	25.81	AMER-S 38.71, AMER-N 29.03, AMER-C 16.13, EURO-W 9.68, CARI 3.23, ASIA-W 3.23,	USA 29.03, COL 29.03, MEX 16.13, BRA 6.45, AUT 6.45, SAU 3.23, PER 3.23, MTQ 3.23, FXX 3.23,
727 (H1) 777737774020731 ▪▪▪▪▪▪▪▪▪▪▪▪□▪▪▪▪▪▪▪▪▪▪▪▪□□□□□□▪□□□□▪▪▪□▪▪▪	777737774020731	13 (3.14)	34.21	AMER-S 68.42, AMER-N 18.42, AFRI-W 5.26	COL 63.16, USA 18.42, NGA 5.26
881 (Unknown) 776377770000731 ▪▪▪▪▪▪▪▪□□▪▪▪▪▪▪▪▪▪▪▪▪▪▪□□□□□□□□□□□□▪▪▪□▪▪▪	776377770000731	6 (1.45)	24	AMER-S 76.0, AMER-N 16.0, EURO-S 8.0,	COL 72.0, USA 16.0, VEN 4.0, ITA 4.0, ESP 4.0,

* Worldwide distribution is reported for regions with more than 3% of a given SITs as compared to their total number in the SITVIT2 database. The definition of macro-geographical regions and sub-regions (http://unstats.un.org/unsd/methods/m49/m49regin.htm) is according to the United Nations; Regions: AFRI (Africa), AMER (Americas), ASIA (Asia), EURO (Europe), and OCE (Oceania), subdivided in: E (Eastern), M (Middle), C (Central), N (Northern), S (Southern), SE (South-Eastern), and W (Western). Furthermore, CARIB (Caribbean) belongs to Americas, while Oceania is subdivided in 4 sub-regions, AUST (Australasia), MEL (Melanesia), MIC (Micronesia), and POLY (Polynesia). Note that in our classification scheme, Russia has been attributed a new sub-region by itself (Northern Asia) instead of including it among rest of the Eastern Europe. It reflects its geographical localization as well as due to the similarity of specific TB genotypes circulating in Russia (a majority of Beijing genotypes) with those prevalent in Central, Eastern and South-Eastern Asia.

** The 3 letter country codes are according to http://en.wikipedia.org/wiki/ISO_3166-1_alpha-3; countrywide distribution is only shown for SITs with ≥3% of a given SITs as compared to their total number in the SITVIT2 database.

**Figure 1:**
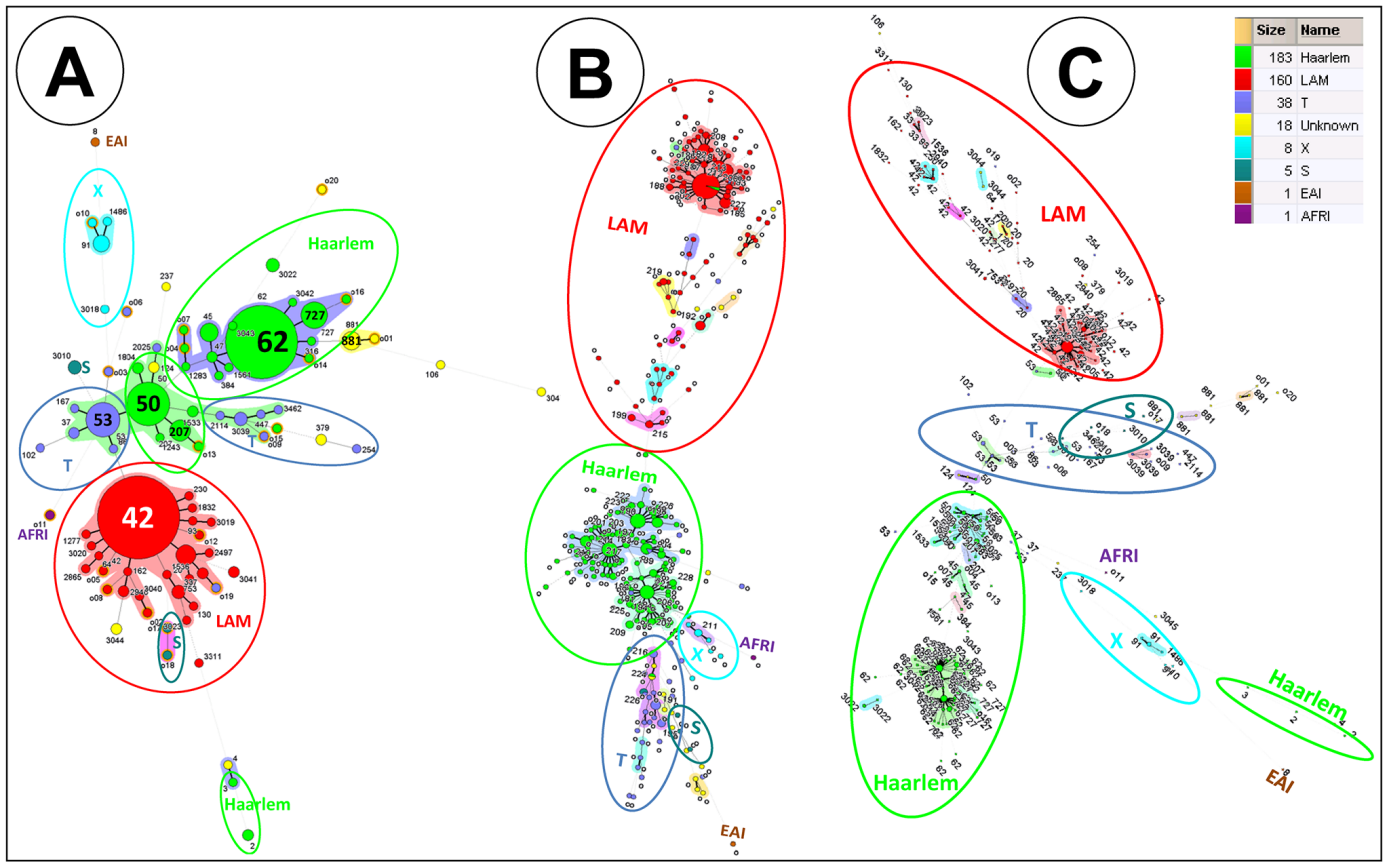
Minimum spanning tree (MST) illustrating evolutionary relationships between *M. tuberculosis* spoligotypes identified in our study (**A**). MST constructed with spoligotyping. (B) MST constructed with 24-locus MIRU-VNTR (**C**) Composite MST with spoligotyping and MIRU-VNTRs markers. MST were constructed on all isolates (n = 414, including 20 orphan patterns). The phylogenetic tree connects each genotype based on degree of changes required to go from one allele to another. The structure of the tree is represented by branches (continuous vs. dashed and dotted lines) and circles representing each individual pattern. Note that the length of the branches represents the distance between patterns while the complexity of the lines (continuous, gray dashed and gray dotted) denotes the number of allele/spacer changes between two patterns: solid lines, 1 or 2 or 3 changes (thicker ones indicate a single change, while the thinner ones indicate 2 or 3 changes); gray dashed lines represent 4 changes; and gray dotted lines represent 5 or more changes. The size of the circle is proportional to the total number of isolates in our study, illustrating unique isolates (smaller nodes) versus clustered isolates (bigger nodes). The color of the circles indicates the phylogenetic lineage to which the specific pattern belongs. Note that orphan patterns are circled in orange. Patterns colored in yellow indicate a strain with an unknown signature (unclassified).

MSTs were constructed based on spoligotype patterns and 24-locus MIRU-VNTRs. Figure 1A shows MSTs based on spoligotypes in which two major groups were evident and belonged to the Haarlem and LAM sublineages, and included most of the isolates (SIT62 and SIT42, respectively, were the most frequent in these lineages). Other isolates were grouped as the ill-defined T sublineage (with SIT 53 as the most frequent) and X sublineage (with SIT91 as the most frequent). More distance was evident among isolates that integrate with the Haarlem sublineage than in those integrating with the LAM sublineage. In contrast, when MSTs were constructed using 24-locus MIRU-VNTR, isolates belonging to LAM appeared more distant than those integrating with the Haarlem sublineage. However, 24-locus MIRU-VNTRs better grouped isolates into major lineages such as LAM, Haarlem, S, T, and X; unique isolates belonging to the African sublineage and East African-Indian sublineage were clearly separated (Figure 1B). MSTs combining spoligotyping and MIRU-VNTR are shown in Figure 1C. There was agreement in the manner in which every genotyping method grouped isolates in the major sublineages. The 24-locus MIRU-VNTR analysis of common SITs (SIT42, SIT62, and SIT50) clearly showed that they are composed of very closely related isolates, which were distinguished by only one or two allele changes. Spoligoforest trees generated by means of the Fruchterman-Reingold algorithm and hierarchical layout (Figure S2 A and B) confirmed the dominance of SIT42 (LAM) and SIT62 (Haarlem). The SIT42 (LAM) cluster was the largest node evolved from SIT53 (T1), from which multiple spoligotypes arose. The second largest spoligotype SIT62 (H1) appears to derive originally from SIT53 (T1) and more recently from SIT50 (H3), finally giving rise to a lower amount of SITs.

An evolutionary MST based on spoligotypes as a function of several associated characteristics showed significant differences between predominant SITs (>2%) and drug resistance (unknown not included) (*p*<0.001) (Figure S1). It is worth noting that all strains belonging to SIT45 (H1) were MDR (6 out of 6). The difference between predominant SITs (>2%) and the three higher *IS6110*-RFLP copy number (8, 9, and 11 bands) was also significant (*p* = 0.011). Significant differences were found between predominant SITs and HIV-positive serology (*p* = 0.044). The proportion of HIV-positive patients was greater among isolates belonging to SIT727 (H1) (2 HIV-positive out of 13) and SIT53 (T1) (2 HIV-positive out of 19)(Figure S1). Unknown HIV-status was not included in the analysis. No significant differences were noted when comparing the sex ratios of all predominant SITs (*p*>0.5).

**Figure 2:**
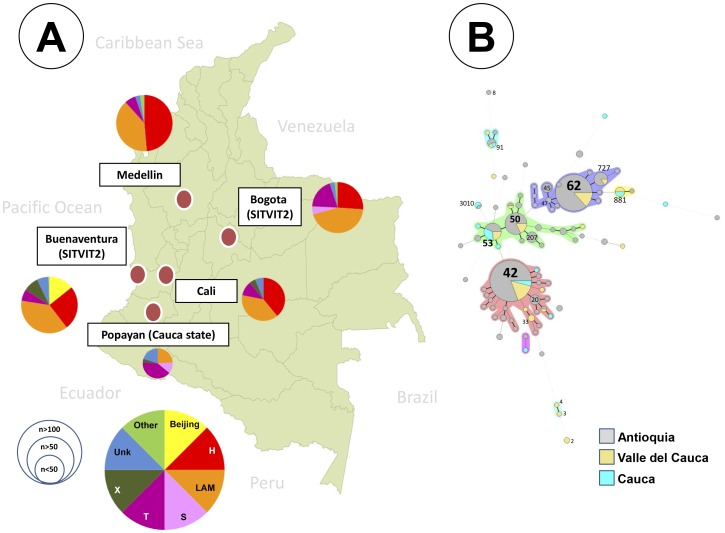
Phylogeographical distribution of *M. tuberculosis* sublineages identified in our study and MST according to patients' origin grouped by cities or states. (**A**) The Map shows the cities of our study and others with their corresponding pie representing the proportion of *M. tuberculosis* sublineages; Distribution of sublineages among strains belonging to the 3 sites of study (**Antioquia**, **Valle del Cauca** and **Cauca**) vs. strains contained in international database SITVIT2 for cities Bogota and Buenaventura. (**B**) MST illustrating evolutionary relationships between *M. tuberculosis* spoligotypes in our study in function of states. The cities have been located into their corresponding states: Medellin is located in **Antioquia**; Cali is located in **Valle del Cauca**; and Popayan, Caldono, Morales, El Tambo, and Piendamo are located in **Cauca state**. The map was obtained from http://www.uxabilidad.com/recursos/mapa-politico-de-colombia-envectores.html which is available as a public domain.

The phylogeographical distribution of *M. tuberculosis* lineages around Colombia is shown in Figure 2A. Our data showed that LAM represented 39.6% of the isolates from Medellín (Antioquia State), 39.1% of the isolates from Cali (Valle state) and 24.0% of the isolates from several towns in Cauca state. The Haarlem sublineage was found to comprise 48.7% of the group of isolates from Medellín and 39.0% of the group of isolates from Cali, but was not represented in isolates from Cauca. In contrast, the ill-defined T sublineage makes up 40.0% of the isolates from Cauca, including the town of Popayan (the main city), as compared to the proportion of isolates from this sublineage in Medellín (6.4%) and Cali (11.0%). No isolates belonging to Beijing sublineage were identified in our study. Genotyping data available in the SITVIT2 database from other two cities in Colombia (Buenaventura and Bogotá), showed a predominance of isolates belonging to the LAM and Haarlem sublineages. Other lineages such as T, X, and S are less represented in these two cities with the exception of the Beijing sublineage which was frequent in the city of Buenaventura.

An MST based on spoligotypes and the state in which the isolates were obtained (Figure 2B) revealed a close evolutionary relationship with the main spoligotypes found in this study, which were LAM (SIT42) and Haarlem sublineages (SIT62) mostly in the states of Antioquia and Valle. In contrast, isolates from Cauca state were more distantly related even among the most frequently identified, the ill-defined T sublineage. The only exception was SIT53 (T1), which was found in greater proportion in Cauca state than in other areas of the country. Furthermore, the difference in the sublineage distribution of isolates from the three states of the country reported in this study was significant (*p*<0.001). The analysis based on 24-locus MIRU-VNTR showed that 52.3% of isolates from Medellin were grouped into 40 clusters (2 to 29 isolates per cluster), 9.4% of isolates from Cali were grouped into 3 clusters (2 isolates each) and 8% of isolates from Popayán and surrounding towns were grouped in one cluster (2 isolates).

Comparative DP was calculated for the three genotyping methods used in this study. The method with the highest DP (0.9916) was 24-locus MIRU-VNTR, followed by *IS6110*-RFLP (0.9868) and then spoligotyping (0.8414). The DP obtained using the combination of the three genotyping methods was slightly higher than that observed for 24-locus MIRU-VNTRs (0.9918 vs. 0.9916). We also evaluated different combinations of MIRU-VNTRs and calculated their corresponding DP. Eight-locus MIRU-VNTR with a allelic diversity greater than 0.6 showed a discriminatory power of 0.9771, while 15-locus MIRU-VNTR with the highest allelic diversity, showed a DP of 0.9855 slightly above of the recommended set of 15-locus MIRU-VNTRs [35], that showed a discriminatory power of 0.9847 (see Table S1). The allelic diversity for the different MIRU-VNTR loci was evaluated using Hunter-Gaston diversity analysis. Locus QUB11b showed the greatest allelic diversity with a diversity index of 0.780 (CI 0.767–0.793), whereas locus 20 showed the lowest diversity index 0.033 (CI 0.009–0.056) (see Table S2).

## Discussion

This study presents a phylogeographic panorama of the *M. tuberculosis* population structure in Colombia. The isolates analyzed from three states showed the LAM and Haarlem clades as being dominant, grouping 82.8% of them mostly in urban settings (Medellín and Cali cities). Other studies carried out in Colombia, based on spoligotyping and deposited in the SITVIT2 database, showed the same predominance of the Haarlem and LAM sublineages. One of these studies was in Bogotá, the major urban setting of Colombia, in which the proportion of the LAM, Haarlem, and T clades were 49.3%, 25.0%, and 13.8% respectively [26]. This study also reported SIT42 (LAM9), SIT62 (H1), and SIT53 (T1) as the major clusters comprising 45.8% of isolates. By contrast, our present study showed the same SITs (42, 62, 53) comprising 58.4% of isolates, of which SIT42 (LAM9) and SIT62 (H1) accounted for 53% of isolates.

The LAM, Haarlem, ill-defined T, X, and S sublineages belong to the Euro-American lineage or lineage 4, one of the six major lineages for *M. tuberculosis* that have been described around the world [2], [36], [37]. This lineage, although present in several regions of the world, is predominant in Europe and America. In Latin America, this lineage is dominant and have been reported by several studies with considerable variation among countries. In our study 37.4% of isolates belonged to LAM. This sublineage appeared to be most common in Brazil (46%), Venezuela (53%), and Peru (28.3%). In the three countries that share borders with Colombia, the proportion of Haarlem is quite variable; our data shows a proportion of 41% among the studied isolates, in contrast to those seen in Venezuela (5%), Brazil (12%), and Peru (28%) [20]–[23], [25].

When comparing the main SITs found in our study with their frequencies in neighboring countries, there is a significant difference in the proportion of SIT42 (LAM9): 29.9% for our study versus 11.8% for Venezuela, 8.8% for Brazil, and 5.6% for Peru (based on the SITVIT2 database). The differences are more striking in the case of SIT62 (H1), which is one of the two most endemic in Colombia, when compared to the same neighboring countries, with 23.4% of isolates belonging to this SIT versus 0.53%, 0.02%, and 0.08% for Venezuela, Brazil, and Peru, respectively (SITVIT2 database) (see Table S3).

Contrary to the sublineage distribution observed in isolates from the main urban settings, those obtained from Cauca state were grouped predominantly (40%) in the ill-defined T sublineage, with no isolates belonging to Haarlem. The clear difference among the distribution of sublineages in Cauca state compared to that in the urban settings of Valle and Antioquia (Cali and Medellín) might be explained by the smaller group of isolates studied, and by the human origin of these isolates. Most of the cases from Cauca state were from patients living in smaller urban and rural areas located in the south of the country, which is characterized by a higher proportion of indigenous population. These facts suggest differences in transmission conditions as well as host factors that ultimately may affect the successful establishment of a particular *M. tuberculosis* lineage in a given human population.

Analysis of *M. tuberculosis* isolates from Buenaventura city (Valle state), a seaport in the South Pacific coast of Colombia, have identified isolates belonging to the LAM and Haarlem sublineages, but also isolates belonging to the Beijing sublineage (SITVIT2 database). This was an unusual finding compared to our study, in which no Beijing isolates were identified. This sublineage, although described for the first time in 1998 in this Colombian seaport city, has only been reported since then from patients whose origin is from this same city, or patients with the same origin but diagnosed in the country's inland major urban settings [38]–[40]. In agreement with these data, despite the human migration from Asia, where the Beijing isolates are very frequent, they represent a proportion about 5% or less of isolates in Latin America, according to several reports [20]–[25].

There was significant association between MDR-TB and HIV status with particular spoligotypes. For example, six isolates belonging to SIT45 (H1) were MDR. Analysis of these isolates using 24-locus MIRU-VNTRs revealed that they were very closely related, but only grouped four of them into two clusters. This finding may represent a particular transmission focus, because all were isolates from patients in Medellín, rather than showing a particular predisposing trend of this spoligotype to develop as MDR. No clear association has been found in terms of predominant lineages or sublineages and MDR among different studies in Latin America [25], [41], [42]. Despite this, the Beijing sublineage has been associated with a high proportion of drug resistant isolates in several parts of the world [43], [44], including Colombia [27]. Moreover, the M strain (Haarlem 2) has been linked to large MDR-TB outbreaks in Argentina [45].

MST analysis provided a detailed picture of genetic distances among *M. tuberculosis* isolates studied based on spoligotyping and MIRUs. Using both genotyping methods facilitated a better assignment of the major and dominant groups belonging to the Euro-American sublineages LAM and Haarlem (lineage 4). This was in agreement with previous studies that demonstrated the utility of this approach in assigning clades and sublineages, particularly within the Euro-American lineage [15]. The hierarchical layout and Fruchterman-Reingold analysis based on spoligotyping interestingly showed that SIT42 (LAM9) and SIT62 (H1), the more conspicuous SITs found in this study, were derived initially from SIT 53 (T1) and lately evolved to the more dominant type. The reason behind the expansion of these particular SITs in the studied isolates and populations, over co-existing non-dominant SITs, might suggest a conjunction of social changes such as accelerated population growth in impoverished sub-urban settings facilitating the transmission of the disease, with mostly still unknown microbe characteristics that allowed the adaptation of particular *M. tuberculosis* isolates to specific human populations. An example of successful *M. tuberculosis* isolates in a particular population was published recently, linking the success of some of them to phenotypic characteristics such as slower growth in monocytes and the ability to elicit a less inflammatory response [46].

A more detailed look at the spoligotyping and 24-locus MIRU-VNTRs composite MST for SIT42 (LAM9) and SIT62 (H1) showed a MIRU-VNTR multiplicity of clusters: 12 clusters in SIT62 and 15 clusters in SIT42, along with unique isolates. Most isolates belonging to these two spoligotypes were very closely related, since they were differentiated by one or two MIRU-VNTR allele changes, supporting the notion that recent expansion and evolution of these groups of isolates have occurred in accordance with the rate of mutation calculated for MIRU-VNTR [17]. The analysis based on 24-locus MIRU-VNTR showed that there was a greater percentage of clustering in isolates from Medellin (52.3%) than in Cali (9.4%) and Popayan and surrounding towns (8.0%). This suggests a more active and ongoing transmission in Medellín (the largest group of isolates studied) than in the other two areas.

A practical utility of the major discriminatory power of the 24-locus MIRU-VNTR set over spoligotyping and *IS6110*-RFLP is its use as an epidemiological marker to distinguish between a diversity of isolates, including those associated with specific transmission chains. This is particularly useful in settings with high endemicity and disease transmission, as observed in one of urban settings studied. Supporting the epidemiological use of MIRU-VNTR in the population studied is the finding that most of the clusters identified by this method were circumscribed to one of the two urban centers studied. In addition, we found that MIRU23, ETRB, and Mtub34, which are excluded from the recommended 15-locus MIRU-VNTR for epidemiological studies [35], had allelic diversity index values higher than 0.5. This finding might lead us to consider the use of these loci in different genotyping studies, especially in areas with *M. tuberculosis* lineage distribution similar to that observed in this study.

In summary, this study shows the distribution of *M. tuberculosis* lineages and sublineages in several regions in Colombia, with an important dominance of LAM and Haarlem belonging to lineage 4, particularly in two major urban settings (Medellín and Cali). Two dominant spoligotypes were LAM9 (SIT42) and Haarlem1 (SIT62). The use of 24-locus MIRU-VNTR showed the best discriminatory power and proved useful in epidemiological studies in which the Euro-American lineage is prevalent. The proportion of the Haarlem sublineage was higher in Colombia compared to that in neighboring countries, suggesting the presence of particular conditions of co-evolution with the corresponding human population that favor the success of this sublineage.

## Supporting Information

Figure S1 **A minimum spanning tree (MST) illustrating evolutionary relationships between the** ***M. tuberculosis*** **spoligotypes in our study in function of studied parameters.** (**A**) Drug resistance; (**B**) *IS6110*-RFLP; (**C**) HIV Serology; (**D**) Sex ratio.Difference between predominant SITs (>2%) including SIT45 vs. Drug resistance (Code 0 Unknown not included) is very significant (p<0.001); note that all strains belonging to SIT45/H1 are MDR. The difference between predominant SITs>2% and the 3 Major *IS6110* RFLP No of Bands (8, 9 and 11) is significant (with a p-value = 0.011). The difference between predominant SITs and HIV serology is significant (p = 0.044), note that the proportion of HIV positive patients is more visible among strains belonging to SIT727/H1 (number of HIV positive = 2/13) and SIT53/T1 (n = 2/19). Missing HIV status values have not been taken into account. No significance difference was observed when comparing sex ratios of all predominant SITs (*p* value>0.5).
(TIF)

Figure S2 **A representation of parent to descendant spoligotypes within our study sample (n = 414 isolates) as seen through Spoligoforest trees drawn using the SpolTools software (available through** http://www.emi.unsw.edu.au/spolTools **; Reyes et al. 2008), and reshaped and colored using the GraphViz software (** http://www.graphviz.org **; J. Ellson et al. 2002).** (**A**) Tree drawn using Fruchterman Reingold algorithm (**B**) tree drawn using a Hierarchical Layout. In both trees, each spoligotype pattern from the study is represented by a node with area size being proportional to the total number of isolates with that specific pattern. Changes (loss of spacers) are represented by directed edges between nodes, with the arrowheads pointing to descendant spoligotypes. The heuristic used selects a single inbound edge with a maximum weight using a Zipf model. Solid black lines link patterns that are very similar, i.e., loss of one spacer only (maximum weigh being 1.0), while dashed lines represent links of weight comprised between 0.5 and 1, and dotted lines a weight less than 0.5. Note that in both trees, SIT42/LAM9 is the biggest node (n = 124, 29.95%), followed by SIT62/H1 (n = 97, 23.43%), SIT50/H3 (n = 34, 8.21%), SIT53/T1 (n = 21, 5.07%) and SIT727/H1 (n = 13, 3.14%), which are other predominant patterns in our study. On the other hand, orphan isolates (double circled), appear mostly at terminal positions on the tree, or are isolated strains without interconnections with the other strains.
(TIF)

Table S1 Comparative discriminatory power of three genotyping methods used in 414 *M. tuberculosis* isolates from Colombia.
(DOCX)

Table S2 Allelic diversity using Hunter-Gaston Diversity index for 24 MIRU-VNTR loci genotyping in 414 *M. tuberculosis* isolates from Colombia.
(DOCX)

Table S3 Distribution of the proportion of predominant SITs in study as compared to their distribution in neighboring countries Venezuela (n = 935), Brazil (n = 4556), and Peru (n = 1296), recorded in the SITVIT2 database.
(DOC)
