# Correction: Population Structure among *Mycobacterium tuberculosis* Isolates from Pulmonary Tuberculosis Patients in Colombia

**DOI:** 10.1371/journal.pone.0109456

**Published:** 2014-09-23

**Authors:** The PLOS ONE Staff

The fourth author’s name is spelled incorrectly. The correct name is: Beatriz Eugenia Ferro.
