# Supplementary figures and images for: Population Structure among Mycobacterium tuberculosis Isolates from Pulmonary Tuberculosis Patients in Colombia
Source: PLoS One. 2014 Apr 18;9(4):e93848. doi: 10.1371/journal.pone.0093848 (PMC3991582; doi:10.1371/journal.pone.0093848)

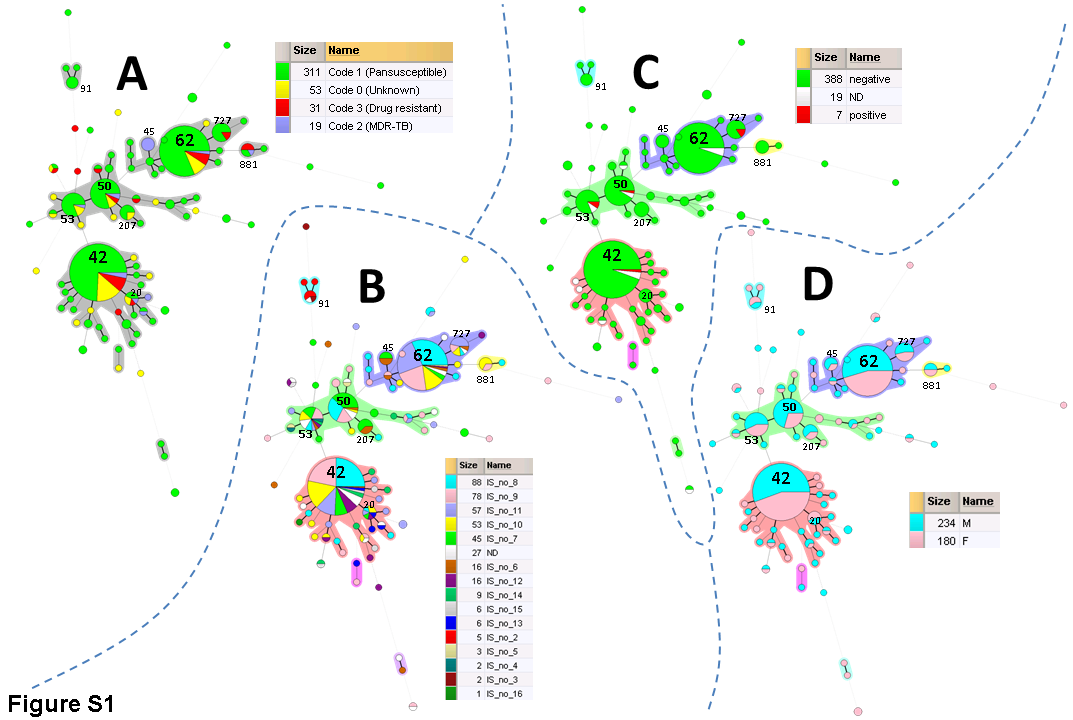

Supplement: Figure S1 — A minimum spanning tree (MST) illustrating evolutionary relationships between the M. tuberculosis spoligotypes in our study in function of studied parameters. (A) Drug resistance; (B) IS6110-RFLP; (C) HIV Serology; (D) Sex ratio.Difference between predominant SITs (>2%) including SIT45 vs. Drug resistance (Code 0 Unknown not included) is very significant (p<0.001); note that all strains belonging to SIT45/H1 are MDR. The difference between predominant SITs>2% and the 3 Major IS6110 RFLP No of Bands (8, 9 and 11) is significant (with a p-value = 0.011). The difference between predominant SITs and HIV serology is significant (p = 0.044), note that the proportion of HIV positive patients is more visible among strains belonging to SIT727/H1 (number of HIV positive = 2/13) and SIT53/T1 (n = 2/19). Missing HIV status values have not been taken into account. No significance difference was observed when comparing sex ratios of all predominant SITs (p value>0.5). (TIF) [file pone.0093848.s001.tif]

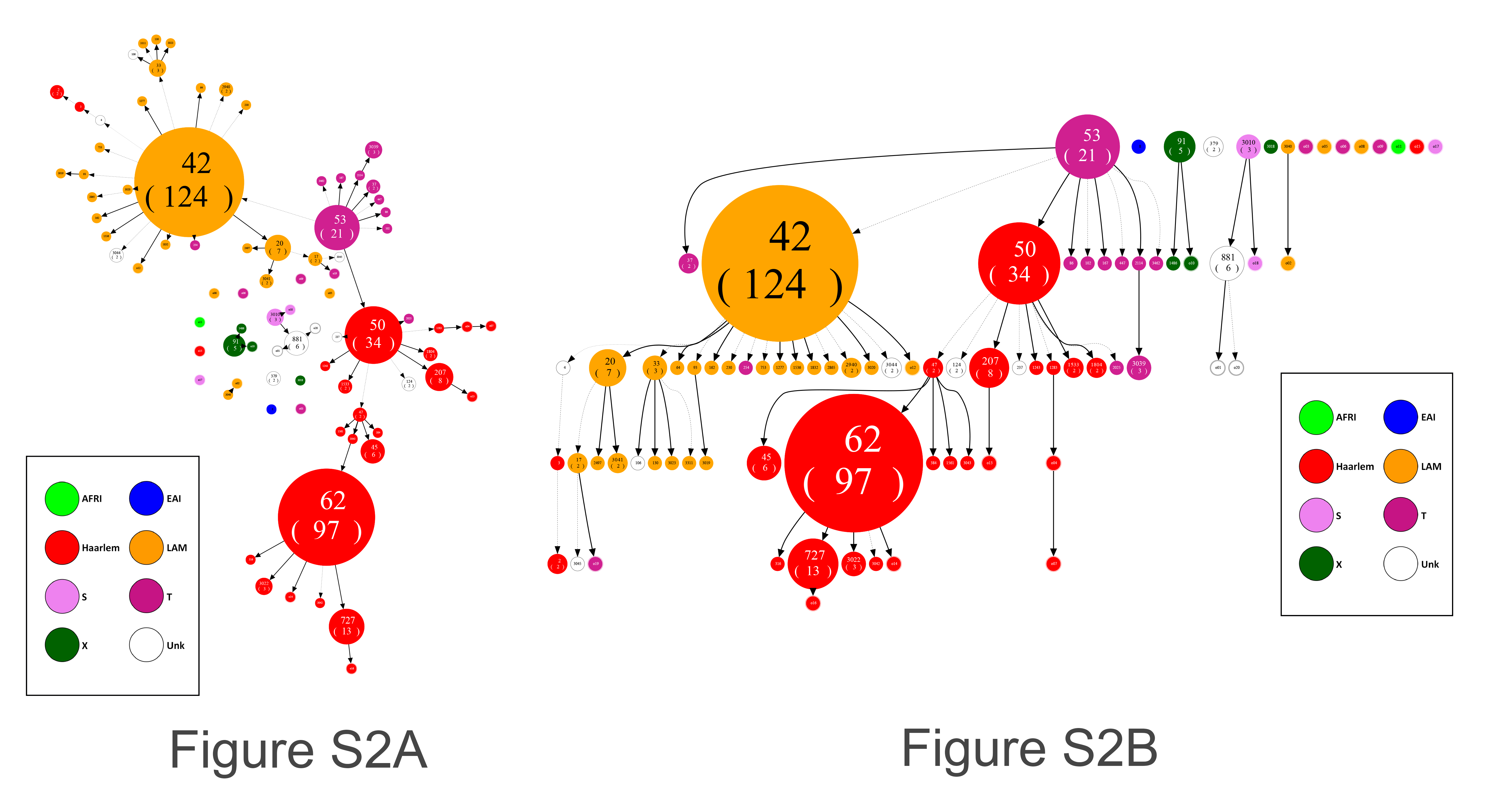

Supplement: Figure S2 — A representation of parent to descendant spoligotypes within our study sample (n = 414 isolates) as seen through Spoligoforest trees drawn using the SpolTools software (available through http://www.emi.unsw.edu.au/spolTools ; Reyes et al. 2008), and reshaped and colored using the GraphViz software ( http://www.graphviz.org ; J. Ellson et al. 2002). (A) Tree drawn using Fruchterman Reingold algorithm (B) tree drawn using a Hierarchical Layout. In both trees, each spoligotype pattern from the study is represented by a node with area size being proportional to the total number of isolates with that specific pattern. Changes (loss of spacers) are represented by directed edges between nodes, with the arrowheads pointing to descendant spoligotypes. The heuristic used selects a single inbound edge with a maximum weight using a Zipf model. Solid black lines link patterns that are very similar, i.e., loss of one spacer only (maximum weigh being 1.0), while dashed lines represent links of weight comprised between 0.5 and 1, and dotted lines a weight less than 0.5. Note that in both trees, SIT42/LAM9 is the biggest node (n = 124, 29.95%), followed by SIT62/H1 (n = 97, 23.43%), SIT50/H3 (n = 34, 8.21%), SIT53/T1 (n = 21, 5.07%) and SIT727/H1 (n = 13, 3.14%), which are other predominant patterns in our study. On the other hand, orphan isolates (double circled), appear mostly at terminal positions on the tree, or are isolated strains without interconnections with the other strains. (TIF) [file pone.0093848.s002.tif]
