# Supplementary material for: Population Structure among Mycobacterium tuberculosis Isolates from Pulmonary Tuberculosis Patients in Colombia
Source: PLoS One. 2014 Apr 18;9(4):e93848. doi: 10.1371/journal.pone.0093848 (PMC3991582; doi:10.1371/journal.pone.0093848)
Supplement: Table S1 — Comparative discriminatory power of three genotyping methods used in 414 M. tuberculosis isolates from Colombia. (DOCX) [file pone.0093848.s003.docx]

**Supplemental table S1**: Comparative discriminatory power of three genotyping methods used in 414 *M. tuberculosis* isolates from Colombia

| Genotyping technique | Discriminatory Power* |
| --- | --- |
| *IS6110*-RFLP | 0.98676138 |
| Spoligotyping | 0.84144049 |
| 24-locus MIRU-VNTR | 0.99156826 |
| *IS6110*-RFLP + Spoligotyping | 0.98956445 |
| *IS6110*-RFLP + 24-locus MIRU-VNTR | 0.99802997 |
| Spoligotyping + 24-locus MIRU-VNTR | 0.99179935 |
| *IS6110*-RFLP + Spoligotyping + 24-locus MIRU-VNTR | 0.9981313 |
| MIRU-VNTR allelic diversity >0.6 (eight) | 0.9771364 |
| Five MIRU-VNTR with best allelic diversity‡ | 0.9635826 |
| MIRU-VNTR with allelic diversity >0. 5 (13)¥ | 0.9844987 |
| Fourteen MIRU-VNTR with best allelic diversityş | 0.9853317 |
| Fifteen MIRU-VNTR with best allelic diversityζ | 0.9855119 |
| Fifteen MIRU-VNTR (Oelemann et al)θ | 0.9846788 |

* Hunter-Gaston discriminatory index

**‡** MIRU10, MIRU40, QUB11b, QUB26, Mtub04.

**¥** MIRU10, MIRU23, MIRU40, ETRA, ETRB, ETRC, QUB11b, QUB26, QUB4156c, Mtub04, Mtub30, Mtub34, Mtub39,.

**ş** MIRU10, MIRU23, MIRU31, MIRU40, ETRA, ETRB, ETRC, QUB11b, QUB26, QUB4156c, Mtub04, Mtub30, Mtub34, Mtub39.

**ζ** MIRU10, MIRU23, MIRU31, MIRU40, ETRA, ETRB, ETRC, QUB11b, QUB26, QUB4156c, Mtub04, Mtub21, Mtub30, Mtub34, Mtub39.

**θ**  MIRU04, MIRU10, MIRU16, MIRU26, MIRU31, MIRU40, ETRA, ETRC, QUB11b, QUB26, QUB4156c, Mtub04, Mtub21, Mtub30, Mtub39.
