# Supplementary material for: Population Structure among Mycobacterium tuberculosis Isolates from Pulmonary Tuberculosis Patients in Colombia
Source: PLoS One. 2014 Apr 18;9(4):e93848. doi: 10.1371/journal.pone.0093848 (PMC3991582; doi:10.1371/journal.pone.0093848)
Supplement: Table S2 — Allelic diversity using Hunter-Gaston Diversity index for 24 MIRU-VNTR loci genotyping in 414 M. tuberculosis isolates from Colombia. (DOCX) [file pone.0093848.s004.docx]

**Supplemental Table S2:** Allelic diversity using Hunter-Gaston Diversity index for 24 MIRU-VNTR loci genotyping in 414 *M. tuberculosis* isolates from Colombia

| Locus | Diversity Index (CI)^1^ | K^2^ | Max(pi)^3^ |
| --- | --- | --- | --- |
| MIRU:QUB11b | 0.780 (0.767 - 0.793) | 7 | 0.263 |
| MIRU:QUB26 | 0.724 (0.697 - 0.750) | 10 | 0.405 |
| MIRU:40 | 0.716 (0.687 - 0.746) | 8 | 0.443 |
| MIRU:10 | 0.690 (0.665 - 0.714) | 5 | 0.403 |
| MIRU:Mtub04 | 0.644 (0.619 - 0.669) | 7 | 0.443 |
| MIRU:Mtub39 | 0.639 (0.615 - 0.663) | 7 | 0.445 |
| MIRU:QUB4156c | 0.637 (0.612 - 0.662) | 7 | 0.495 |
| MIRU:Mtub30 | 0.610 (0.588 - 0.631) | 6 | 0.462 |
| MIRU:ETRA | 0.575 (0.552 - 0.598) | 5 | 0.495 |
| MIRU:Mtub34 | 0.564 (0.535 - 0.593) | 6 | 0.550 |
| MIRU:23 | 0.540 (0.516 - 0.565) | 8 | 0.552 |
| MIRU:ETRC | 0.528 (0.513 - 0.543) | 4 | 0.500 |
| MIRU:ETRB | 0.515 (0.477 - 0.553) | 5 | 0.633 |
| MIRU:31 | 0.462 (0.427 - 0.498) | 5 | 0.666 |
| MIRU:Mtub21 | 0.445 (0.399 - 0.491) | 6 | 0.709 |
| MIRU:02 | 0.419 (0.382 - 0.456) | 3 | 0.706 |
| MIRU:16 | 0.316 (0.265 - 0.367) | 4 | 0.813 |
| MIRU:26 | 0.294 (0.238 - 0.351) | 8 | 0.836 |
| MIRU:27 | 0.122 (0.080 - 0.164) | 3 | 0.936 |
| MIRU:04 | 0.101 (0.061 - 0.140) | 8 | 0.948 |
| MIRU:Mtub29 | 0.083 (0.047 - 0.120) | 5 | 0.957 |
| MIRU:24 | 0.042 (0.015 - 0.069) | 4 | 0.979 |
| MIRU:39 | 0.042 (0.015 - 0.069) | 3 | 0.979 |
| MIRU:20 | 0.033 (0.009 - 0.056) | 2 | 0.983 |

^1^Variation of the number of repeats at each locus. Ranges from 0.0 (no diversity) to 1.0 (complete diversity). ^2^Number of different repeats present at this locus in the group of isolates studied. ^3^Fraction of samples that have the most frequent repeat number in this locus (range 0.0 to 1.0).
