# Supplementary material for: Population Structure among Mycobacterium tuberculosis Isolates from Pulmonary Tuberculosis Patients in Colombia
Source: PLoS One. 2014 Apr 18;9(4):e93848. doi: 10.1371/journal.pone.0093848 (PMC3991582; doi:10.1371/journal.pone.0093848)
Supplement: Table S3 — Distribution of the proportion of predominant SITs in study as compared to their distribution in neighboring countries Venezuela (n = 935), Brazil (n = 4556), and Peru (n = 1296), recorded in the SITVIT2 database. (DOC) [file pone.0093848.s005.doc]

|  |  |  |  |  |  | **Distribution in neighboring countries** | | | | | |
| --- | --- | --- | --- | --- | --- | --- | --- | --- | --- | --- | --- |
| **SIT** | **Spoligotype Description** | **Octal Code** | **This study n/t (%)** | **COLOMBIA without study n/t (%)** | **p-value** | **VENEZUELA n/t (%)** | **p-value** | **BRAZIL**  **n/t (%)** | **p-value** | **PERU**  **n/t (%)** | **p-value** |
| 20 | ■■□■■■■■■■■■■■■■■■■■□□□□■■■■■■■■□□□□■■■■■■■ | 677777607760771 | 7/414 (1.69) | 6/432 (1.39) | 0.7212 | 42/935 (4.49) | 0.0112* | 147/4556 (3.23) | 0.0843 | 5/1296 (0.39) | 0.0056* |
| 42 | ■■■■■■■■■■■■■■■■■■■■□□□□■■■■■■■■□□□□■■■■■■■ | 777777607760771 | 124/414 (29.95) | 93/432 (21.53) | 0.005* | 111/935 (11.87) | <0.0001* | 401/4556 (8.80) | <0.0001* | 73/1296 (5.63) | <0.0001* |
| 45 | ■■■■■■■■■■■■■■■■■■■■■■■□■□□□□□□■□□□□■■■■■■■ | 777777764020771 | 6/414 (1.45) | 0/432 (0.00) | 0.013* | 0/935 (0.00) | <0.001* | 9/4556 (0.20) | <0.0001* | 0/1296 (0.00) | <0.0002* |
| 50 | ■■■■■■■■■■■■■■■■■■■■■■■■■■■■■■□■□□□□■■■■■■■ | 777777777720771 | 34/414 (8.21) | 14/432 (3.24) | 0.0018* | 13/935 (1.39) | <0.0001* | 239/4556 (5.25) | 0.0112* | 160/1296 (12.35) | 0.0210* |
| 53 | ■■■■■■■■■■■■■■■■■■■■■■■■■■■■■■■■□□□□■■■■■■■ | 777777777760771 | 21/414 (5.07) | 28/432 (6.48) | 0.3805 | 49/935 (5.24) | 0.8978 | 322/4556 (7.07) | 0.1252 | 118/1296 (9.10) | 0.009* |
| 62 | ■■■■■■■■■■■■■■■■■■■■■■■■■□□□□□□■□□□□■■■□■■■ | 777777774020731 | 97/414 (23.43) | 68/432 (15.74) | 0.0048* | 5/935 (0.53) | <0.0001* | 1/4556 (0.02) | <0.0001* | 1/1296 (0.08) | <0.0001* |
| 91 | ■■■□□□□□□□□□□■■■■□■■■■■■■■■■■■■■□□□□■■■■■■■ | 700036777760771 | 5/414 (1.21) | 7/432 (1.62) | 0.6119 | 2/935 (0.21) | 0.0314* | 4/4556 (0.09) | <0.001* | 25/1296 (1.93) | 0.3305 |
| 207 | ■■■■■□■■■■■■■■■■■■■■■■■■■■■■■■□■□□□□■■■■■■■ | 767777777720771 | 8/414 (1.93) | 0/432 (0.00) | 0.0032* | 0/935 (0.00) | <0.0001* | 2/4556 (0.04) | <0.0001* | 1/1296 (0.08) | <0.0001* |
| 727 | ■■■■■■■■■■■■□■■■■■■■■■■■■□□□□□□■□□□□■■■□■■■ | 777737774020731 | 13/414 (3.14) | 10/432 (2.31) | 0.4606 | 1/935 (0.11) | <0.0001* | 0/4556 (0.00) | <0.0001* | 0/1296 (0.00) | <0.0001* |
| 881 | ■■■■■■■■□□■■■■■■■■■■■■■■□□□□□□□□□□□□■■■□■■■ | 776377770000731 | 6/414 (1.45) | 10/432 (2.31) | 0.3555 | 1/935 (0.11) | 0.0042* | 0/4556 (0.00) | <0.0001* | 0/1296 (0.00) | <0.0002* |

**Supplemental Table S3**: Distribution of the proportion of predominant SITs in study as compared to their distribution in neighboring countries Venezuela (n=935), Brazil (n=4556), and Peru (n=1296), recorded in the SITVIT2 database.

* Asterisk denotes statistically significant differences (p<0.05); Pearson's Chi-square test was used when more than 80% of data had a value greater than 5, and Fisher's Exact Test for remaining data with smaller values (at least 20% of data having values less than 5).
